# Exploring the views of university students with experience of common mental health disorders about support provided within primary care community settings

**DOI:** 10.1192/bjo.2021.105

**Published:** 2021-06-18

**Authors:** Mikaela D'Arcy-Smith, Marta Buszewicz

**Affiliations:** 1 UCL Medical School; 2 Honorary Associate Professor - UCL Department of Clinical, Educational and Health Psychology, University College London

## Abstract

**Aims:**

To assess the impact of common mental health disorders (CMHDs) on university students’ function and wellbeing. To understand the barriers to receiving adequate support for CMHDs during both adolescence and at university. To provide feedback to healthcare professionals about how young people perceive the support provided when initially seeking help for psychological distress. To explore which forms of support students find the most useful.

**Method:**

A literature review was initially undertaken, identifying the lack of prior research in this area. The current study addressed the gap by considering the needs of students with CMHDs in the context of primary care services, with a retrospective exploration of their views about support received during adolescence. 15 semi-structured qualitative interviews were conducted with both current university students and recent graduates from across the UK, transcribed verbatim and subjected to thematic analysis. The study population included 7 men and 8 women, between the ages of 18–25 years.

**Result:**

Five main themes emerged from the data:

The Journey to Disorder – Explored the difficulties faced by adolescents, and how these might contribute to their experience of CMHDs and their management.

Attitudes Towards Help-Seeking – Many participants had little trust in healthcare professionals as adolescents. This contributed to limited trust in university support systems as young adults.

Primary Care Support - Perceived effectiveness of General Practitioner (GP) support during adolescence in this cohort was highly variable. Although some participants described good experiences, others felt their views were ignored, with responsibility diverted to their caregivers. A lack of understanding from GPs about CMHDs in adolescents resulted in trust issues for them as young adults.

Recommendations for Change - Participants reflected on their previous and current experiences to inform suggestions for changes to tackle issue of psychological distress in adolescents.

**Conclusion:**

Previous experiences of the care they had received when presenting with CMHDs during adolescence potentially affected the long-term wellbeing of university students and graduates; the initial support received was inconsistent with the needs of this age group. Recommendations for change included a greater emphasis on the importance of adolescent mental health education, tailoring interventions to personal growth and maturity, and ensuring primary healthcare providers are equipped with the skills required to manage psychological distress in young people.

